# Surface Modification of Biodegradable Mg-Based Scaffolds for Human Mesenchymal Stem Cell Proliferation and Osteogenic Differentiation

**DOI:** 10.3390/ma14020441

**Published:** 2021-01-18

**Authors:** Si-Han Wang, Shiao-Pieng Lee, Chung-Wei Yang, Chun-Min Lo

**Affiliations:** 1 Department of Biomedical Engineering, National Yang-Ming University, Taipei 11221, Taiwan; d40404006@ym.edu.tw; 2 Division of Oral and Maxillofacial Surgery, Department of Dentistry, School of Dentistry, Tri-Service General Hospital, National Defense Medical Center, Taipei 11490, Taiwan; shiao-pieng@yahoo.com.tw; 3 Department of Materials Science and Engineering, National Formosa University, Yunlin 632, Taiwan

**Keywords:** magnesium, biodegradation, fluorohydroxyapatite, human mesenchymal stem cell, bone tissue engineering

## Abstract

Magnesium alloys with coatings have the potential to be used for bone substitute alternatives since their mechanical properties are close to those of human bone. However, the surface modification of magnesium alloys to increase the surface biocompatibility and reduce the degradation rate remains a challenge. Here, FHA-Mg scaffolds were made of magnesium alloys and coated with fluorohydroxyapatite (FHA). Human mesenchymal stem cells (hMSCs) were cultured on FHA-Mg scaffolds and cell viability, proliferation, and osteogenic differentiation were investigated. The results showed that FHA-Mg scaffolds display a nano-scaled needle-like structure of aggregated crystallites on their surface. The average Mg^2+^ concentration in the conditioned media collected from FHA-Mg scaffolds (5.8–7.6 mM) is much lower than those collected from uncoated, Mg(OH)_2_-coated, and hydroxyapatite (HA)-coated samples (32.1, 17.7, and 21.1 mM, respectively). In addition, compared with hMSCs cultured on a culture dish, cells cultured on FHA-Mg scaffolds demonstrated better proliferation and comparable osteogenic differentiation. To eliminate the effect of osteogenic induction medium, hMSCs were cultured on FHA-Mg scaffolds in culture medium and an approximate 66% increase in osteogenic differentiation was observed three weeks later, indicating a significant effect of the nanostructured surface of FHA-Mg scaffolds on hMSC behaviors. With controllable Mg^2+^ release and favorable mechanical properties, porous FHA-Mg scaffolds have a great potential in cell-based bone regeneration.

## 1. Introduction

Autologous bone grafting is a common approach to replace missing bone or to repair bone fractures. However, the clinical use of autologous bone grafting is constrained by size limitations and donor site morbidity. Artificial bone tissue is therefore desired to be used as a permanent implantation [1]. Bone tissue engineering involves the use of cells, biochemical factors, and scaffolds to provide the structure to support recovery and regeneration time [2,3]. In the use of orthopedic implants, Mg and its alloys have caught our attention because they are biodegradable and can enhance new bone formation while maintaining desired mechanical properties during bone recovery [4,5].

The Young’s moduli of Mg-based alloys (41–45 GPa) are comparable to those of human bone (10–40 GPa) when compared with the Young’s moduli of commercially used Ti-based alloys or stainless steel [6]. Over the last decade, Mg and several Mg-based alloys, such as Mg-Al-Zn, LAE442, WE43, and Mg-Zn alloys, have been investigated and developed as different components for biodegradable metallic materials [4,7,8,9,10]. Surface modification technologies have been applied to construct multi-functional surfaces of metallic biomaterials [11,12], which exhibit excellent biocompatibility and bioactivity and can be used as bone substitutes in the form of particles, blocks, and coatings [12,13,14,15]. For example, the pore size and porosity of Mg scaffolds with controllable microstructures promote osteoblast proliferation and differentiation [16]. The increase in the surface roughness of hydroxyapatite (HA) coating enhances the specific adsorption of serum proteins and further increases human bone marrow cell adhesion and proliferation [17,18]. In addition, porous HA-coated metals have been shown to enhance cell viability and degrade much slower than those that are uncoated [19].

Another advantage of using Mg-based scaffolds for tissue engineering is their subsequent degradation and Mg^2+^ release during the formation of new tissues [6,20]. Of importance is the fact that Mg-based scaffolds can be fully degraded after tissue regeneration, which satisfies the increasing demand for better biomedical devices and functional biomaterials in tissue engineering [21]. However, Mg-based implants with the desired degradation rates remain a challenge because they are chemically active in physiological environments. It has been reported that both low and high magnesium concentrations have harmful effects on bones, while moderate magnesium concentrations are able to support the healing process of diseased or damaged tissues [22,23]. Mg^2+^ can improve bone mineral density and bone fragility [24,25]. A lack of Mg^2+^ influences all stages of skeletal metabolism, retards the bone growth, and results in osteoporosis [26,27]. A high Mg^2+^ concentration leads to mineralization defects which are possibly due to the partial substitution of Ca element by Mg within the crystal structure of HA [28]. A slower release of Mg^2+^ from scaffolds can contribute to bone regeneration in vivo [29,30], whereas a hyper-physiological level of Mg^2+^ concentration inhibits extracellular matrix formation and supports chondrocyte proliferation [31].

Since the concentration of Mg^2+^ ions may influence bone remodeling and/or cause cytotoxicity [32,33], a controllable and protective coating is particularly needed for regulating the degradation rate of Mg alloys [29,34]. Moreover, a surface coating may also help to prevent the unwarranted overload of released hydrogen bubbles in human metabolism while maintaining the mechanical integrity of the implants, allowing them to remain intact before the adequate restoration of new tissues. Calcium phosphate (CaP) coatings, such as dicalcium phosphate dihydrate (DCPD), tricalcium phosphate (TCP), and hydroxyapatite (HA), have been suggested as a means to control the degradation rates of biodegradable magnesium and its alloy [12,35]. These CaP coatings demonstrate excellent biocompatibility and osteoconduction since Ca and P are the main elements in bone minerals. In particular, HA has a chemical and structural resemblance to natural bone and has been widely used as the coating material for orthopedic and dental endosseous implants. However, HA coatings on the surface of Mg alloy suffer from a relatively high dissolution rate in bodily fluids, which decreases the long-term stability of the implants [36,37]. Recently, the incorporation of fluorine into HA (FHA: Ca_10_(PO_4_)_6_(OH)_2–x_F_x_) has been demonstrated to effectively decrease the dissolution rate [13,38,39,40,41,42]. It was reported that FHA coatings enhance osteoblastic proliferation and osteogenic differentiation [43]. Furthermore, in vivo experiments showed that FHA has an inhibited effect on osteoclastic activity and suppresses bone absorption [44]. Thus, fluoridated hydroxyapatite holds great potential for the functional coating of biodegradable magnesium implants [45].

Despite the fact that several FHA synthesis techniques, such as precipitation, hydrolysis, hydrothermal, sol–gel, and electrodeposition methods, have been developed, there remain many critical factors to be explored in this field [15,46]. Previously, we applied a hydrothermal synthesizing process to deposit uniform fluorine-substituted HA (FHA) coatings on the Mg-8.5Al-0.5Zn (AZ80) Mg alloy [13]. This coating was composed of a 200 μm Mg(OH)_2_ intermediate layer and a 50 μm HA/FHA top coat. Needle-like crystals formed on the surface of the FHA coating after synthesis. The analyzed results showed that fluorine ions were successfully substituted into the HA crystal structure and the corrosion resistance of AZ80 in Kokubo’s simulated body fluid was effectively improved. Compared with the HA coating, the higher corrosion resistance of FHA coatings results from its smaller Ca/P ratio and dense microstructure [13]. In this study, a hydrothermal synthesis technique was applied to coat an FHA nanocomposite on AZ91 Mg alloys and their porosity, surface roughness, and corrosion characteristics were examined. Human mesenchymal stem cells (hMSCs) were used to investigate the effects of the FHA coating on cell viability, proliferation, and osteogenic differentiation. Our ultimate goal is to elucidate the possible use of FHA-Mg materials for cell-based bone regeneration and further medical application [46].

## 2. Materials and Methods

### 2.1. Surface Coating of Mg-Based Scaffolds and Collection of Mg^2+^ Conditioned Media

The base metal used in this study was a 3 mm thick Mg-Al-Zn sheet with a chemical composition of 8.8 Al, 0.7 Zn, 0.22 Mn, 0.02 Si, and Mg balance (in wt.%, named AZ91, Pinda Technology Co., Ltd., Taiwan), which was determined by inductively coupled plasma–atomic emission spectrometry (ICP/AES). These specimens with dimensions of 10 (L) × 10 (W) × 3 (T) mm^3^ were prepared as substrates for the hydrothermal synthesis of surface coatings. Analytical grade dicalcium phosphate dehydrate (DCPD, CaHPO_4_·2 H_2_O), calcium hydroxide (Ca(OH)_2_), and hexafluorophosphoric acid (~55 wt.% HPF_6_ in H_2_O, Sigma-Aldrich) were used as reactants. First, a suspension containing powdered mixtures of DCPD and Ca(OH)_2_ with deionized water was prepared. The aqueous solution with a controlled Ca/P molar ratio of 1.67 was used for hydrothermally synthesizing HA coatings on the AZ91 substrates, and these specimens were denoted by “HA-Mg”. As for the fabrication of fluorine-substituted HA (fluorohydroxyapatite, FHA) coatings, the substitution content of F¯ ions instead of OH¯ groups was determined by the x value in the formula of Ca_10_(PO_4_)_6_(OH)_2–x_F_x_ [47]. Next, 3 M of HPF_6_ was added into the DCPD/Ca(OH)_2_ mixture to obtain an aqueous solution, where the Ca/P molar ratio was also controlled as 1.67. The hydrothermally synthesized FHA-coated specimens were labeled as “FHA-Mg”. The final mixed solutions with a pH of 12 were used as the reagents for hydrothermally synthesizing HA and FHA coatings on the AZ91 Mg alloy. The chemical composition of the hydrothermally synthesized FHA coating is Ca_10_(PO_4_)_6_(OH)F, which is the optimal condition based on our previous study [13]. The surface coating of Mg(OH)_2_ was also synthesized on the AZ91 alloy at 175 °C by the hydrothermal method. Both HA and FHA solutions of 300 mL were poured into the autoclave. Next, AZ91 substrates were directly immersed in the deionized water, and the autoclave was heated to hydrothermal temperature. These specimens were labeled as “Mg(OH)_2_-Mg”. The hydrothermal synthesis process for the abovementioned HA-Mg, FHA-Mg, and Mg(OH)_2_-Mg coatings was performed at 175 °C and held for 2 h in a hermetical autoclave (Parr 4621). Conditioned culture and differentiation media were prepared with Mg-based scaffolds incubated in culture media and in osteogenic induction media, respectively, at a weight ratio of 0.2 g/mL, according to EN ISO standards ISO 10993-5 and 10993-12 [48,49]. For all the FHA-Mg, HA-Mg, Mg(OH)_2_-Mg, and uncoated Mg scaffolds, each 1 g of sample was placed in a 6-well plate with 5 mL of culture/differentiation medium in the well and incubated in a 5% CO_2_ incubator at 37 °C. The immersed conditioned medium was collected and replaced with 5 mL of fresh medium every 3 days. Conditioned medium was collected in this way for 30 days from each sample incubation, filtered through a 0.22 μm membrane, and labeled (Table 1). The Mg^2+^ concentration and pH value of each conditioned medium were analyzed with ICP-AES and a pH meter (JENCO Electronics 6173 pH).

### 2.2. Surface Roughness of Mg-Based Scaffold

The surface roughness of the coating was evaluated using a profilometer (Surfcorder SE1200, Kosaka). Specimens were cross-sectioned with a low-speed diamond saw and mounted in epoxy resin. The mounted specimens were carefully ground and polished to avoid inducing extra pores and cracks. The porosity content (in volume %) of the coating was then quantitatively analyzed using an optical microscope equipped with OPTIMAS 6.1 image-analyzing software (Optimas Corporation, Bothell, WA, USA). Basically, a 100 square millimeter area was selected on the coating, and the image was taken and analyzed. The same procedure was repeated at 3 random locations to obtain the average porosity percentage.

### 2.3. Cell Culture

Human mesenchymal stem cells (hMSCs) isolated from Wharton’s jelly of the umbilical cord were purchased from the Bioresource Collection and Research Center (BCRC, No. RM60596), Hsinchu, Taiwan. Cells were cultured in Dulbecco’s modified Eagle’s medium (Invitrogen, CA, USA) supplemented with 2% fetal bovine serum (Thermo), epidermal growth factor (PeproTech), platelet-derived growth factor (PeproTech), dexamethasone (Sigma), and L-ascorbic acid-2-phosphate (Sigma) under 5% CO_2_ at 37 °C. Only hMSCs passaged 7–10 were used in our experiments. Before inoculating cells, Mg-based scaffolds were immersed in culture medium for 9 days to remove the large amount of initially released Mg^2+^ ions. Following that, approximately 4 × 10^5^ hMSCs were seeded onto each Mg-based scaffold, which was placed in a 12-well polystyrene plate, and stored at 37 °C in a humidified incubator with 5% CO_2_. To induce osteogenic differentiation, hMSCs were treated with osteogenic induction medium for 21 days and the medium was changed every 3 days. The osteogenic induction medium was Dulbecco’s Modified Eagle Medium (DMEM) (Invitrogen, Carisbad, CA, USA) supplemented with 10% fetal bovine serum (FBS) (Thermo Fisher Scientific, Waltham, MA, USA), 0.1 μM of dexamethasone (Sigma-Aldrich, St. Louis, MO, USA), 50 μM of L-ascorbic acid-2-phosphate (Sigma-Aldrich, St. Louis, MO, USA), and 10 mM of β-glycerophosphate disodium (Sigma-Aldrich, USA).

### 2.4. Scanning Electronic Microscopy (SEM)

The surface morphologies of Mg(OH)_2_, HA, and FHA scaffolds were examined by scanning electron microscopy (SEM, HR FESEM, JSM-7600F, JEOL, Japan). Cells cultured on Mg-based scaffolds were rinsed with phosphate buffer and then fixed in 2.5% glutaraldehyde for 2 h at room temperature. After being rinsed with phosphate buffer, humid Mg-based scaffolds were dehydrated with serial concentrations of ethanol, at 50%, 70%, 90%, and 95%, for 10 min each and finally with 100% ethanol for 10 min three times. By using the critical point dryer (CPD) (PVT-3B, Tousimis, Rockville, MD, USA), the ethanol was replaced with liquid CO_2_ and the chamber was sealed and heated until the critical point of CO_2_. Dehydrated samples were fixed on standard SEM copper stubs by carbon tapes, sputter-coated with gold, and then examined by SEM.

### 2.5. Cell Viability and Proliferation Assays

Cell viability in Mg^2+^ conditioned media was evaluated by 3-(4,5-Dimethylthiazol-2-yl)-2,5-diphenyltetrazolium bromide (MTT) assay. First, 3 × 10^3^ cells were seeded onto each well of the 96-well plates in hMSC culture medium. After cells attached, the medium was changed to Mg^2+^ conditioned culture medium and the cells were cultured in the conditioned medium for 2 days. Subsequently, 200 μL of the 3-(4,5-dimethylthiazol-2-yl)-2,5-diphenyltetrazolium bromide (MTT) working solution were added into each well and incubated for 4 h. The absorbance of the converted dye was measured at a fixed emission wavelength of 570 nm in an enzyme-linked immunosorbent assay (ELISA) reader (Tecan, Sunrise). Relative cell viability was evaluated by the optical density (OD) value of each sample and compared with the control. For cell viability assessment, experiments were repeated 3 times with 8 replicates (*n* = 8) in conditioned media A to J (Table 1) collected from different Mg-based scaffolds. Cell proliferation in FHA-Mg conditioned media was evaluated by an alamarBlue assay (Invitrogen). After cells attached, the complete culture medium was changed to conditioned culture medium. At each culturing stage (days 1, 3, and 5), 10% alamarBlue solution was added into wells and incubated at 37 °C for 6 h. Next, 150 μL sample solution were transferred to 96-well plates to measure the absorbance at an OD of 570/600 nm.

### 2.6. Cell Differentiation Assay

To quantify osteogenic differentiation of hMSCs, alkaline phosphatase (ALP) activity was assessed using an ALP activity colorimetric assay kit (BioVision, Milpitas, CA, USA). In the ALP assay, 50 μL of 5 mM p-nitrophenyl phosphate (pNPP) as a phosphatase substrate were added into each well containing 80 μL of the culture supernatant. The reaction was at room temperature for 1 h in the dark. To test both sample and background controls, the reaction was stopped by adding 20 μL of 0.2 N NaOH and OD was measured at 405 nm in a microplate reader. The osteogenic differentiation of hMSCs cultured on FHA-Mg scaffolds and culture dishes in the presence of osteogenic induction medium or complete culture medium was compared at days 0, 7, 14, and 21.

### 2.7. Quantitative Polymerase Chain Reaction (qPCR)

The expression of ALP and osteocalcin (OCN) mRNA was determined by qPCR on day 14. The total cell mRNA obtained from each scaffold (*n* = 3) was harvested using TRIzol reagent (Invitrogen Life Technologies). cDNA was transcribed from 1 μg of total RNA, using the SensiFAST^TM^ cDNA synthesis kit (Bioline, UK) and following the manufacturer’s protocol. Reactions were performed in a final volume of 20 μL, using 4 μL of buffer and 1 μL of reverse transcriptase provided by the supplier. The SensiFAST^TM^ SYBR Hi-ROX System (Bioline, UK) was used for qPCR. One microliter of cDNA was mixed with 10 μL of 2× SensiFAST SYBR Hi-ROX Mix, 0.8 μL of 10 μM forward primer (400 nM final concentration), 0.8 μL of 10 μM reverse primer (400 nM final concentration), and nuclease-free water to 20 μL. The sequences of primers (Genomics, Taiwan) used are listed in Table 2. A 3-step cycling was used on a Bio-Rad CFX96 instrument: 1 cycle of 95 °C for 2 min to activate the polymerase, followed by 40 cycles of 95 °C for 5 s to denaturation, 65 °C for 10 s for annealing, then 10 s at 72 °C for extension. Relative gene expression levels of ALP and OCN were normalized to the expression of the reference gene glyceraldehyde 3-phosphate dehydrogenase (GAPDH). Each PCR was duplicated with the same amount of total mRNA. The relative expression of each target gene was evaluated via the 2^−ΔΔCT^ method and analyzed by ABI StepOne Plus V2.3 Software (Applied Biosystems, Waltham, MA, USA).

### 2.8. Statistical Analysis

Statistical analysis was performed using a Student’s *t*-test. The level of significance was set at * *p* < 0.05, ** *p* < 0.01, and *** *p* < 0.001. All data are expressed as mean ± standard error.

## 3. Results

### 3.1. Structure and Surface Characterization of Hydrothermal Mg-Based Scaffolds

**Figure 1:**
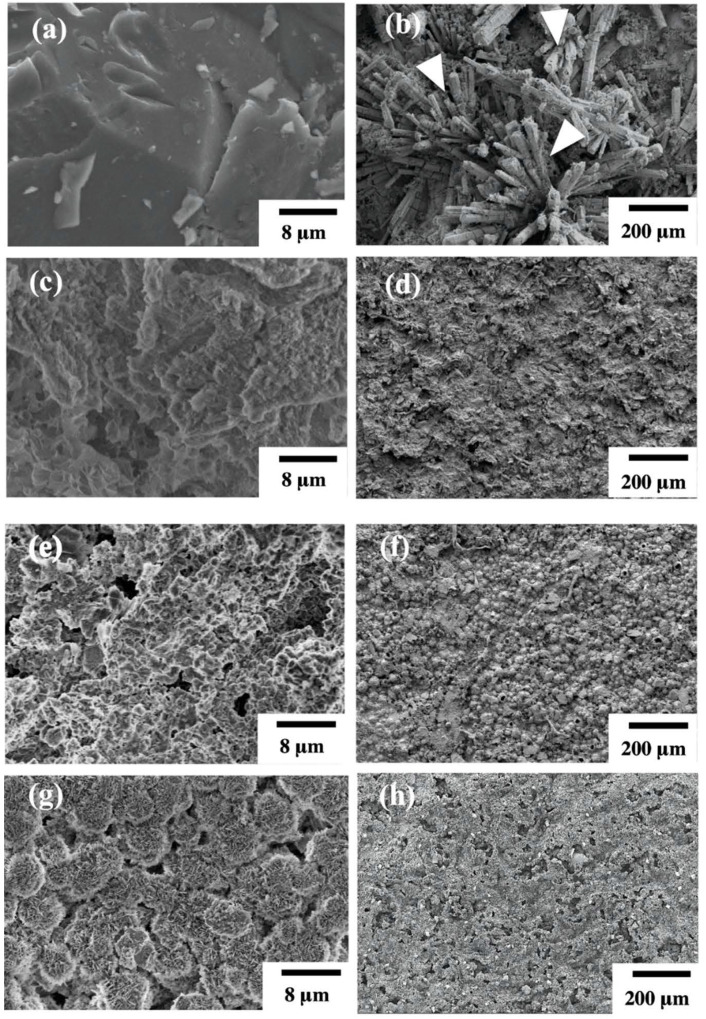
Surface morphologies of the uncoated Mg scaffold (**a**) before incubation (3000× magnification) and (**b**) after incubation (100× magnification). Surface morphologies of hydrothermally coated (**c**) Mg(OH)_2_-Mg, (**e**) HA-Mg, and (**g**) FHA-Mg scaffolds before incubation and (**d**,**f**,**h**) after incubation for 9 days.

Figure 1 shows the SEM surface microstructures of uncoated Mg scaffolds and hydrothermally coated Mg(OH)_2_-Mg, HA-Mg, and FHA-Mg scaffolds. Figure 1a is a typical surface morphology of grit-blasted uncoated Mg scaffold with a surface roughness of about 0.09 ± 0.03 μm. It reveals that surface grooves are present on the AZ91 alloy after the grit-blasting process. After the hydrothermal synthesis process, Mg(OH)_2_-Mg, HA-Mg, and FHA-Mg scaffolds display different surface morphologies, as illustrated in Figure 1c,e,g. It is worth noting that nano-scaled needle-like aggregated crystals are observed on the surface of the FHA-Mg scaffold (Figure 1g). The formation and detail clarification of FHA-Mg surface features have been described in our previous study [13]. After incubation in culture medium for several days, a large amount of columnar calcium phosphate compounds (as indicated by the arrow in Figure 1b) is observed on the grit-blasted uncoated Mg scaffold. This corrosion process probably involved the release of hydrogen and environmental alkalinization, leading to the formation of corrosion products such as MgO and Mg(OH)_2_. However, the differences in the surface morphologies of Mg(OH) _2_-Mg, HA-Mg, and FHA-Mg scaffolds are unapparent (Figure 1d,f,h). Table 3 lists the measured surface roughness, porosity, and pore size of the four specimens. Coated FHA-Mg scaffolds with a nano-scaled needle-like aggregated microstructure display the smallest surface roughness. Since the average diameter of hMSCs is about 18–30 μm [50], the hydrothermally coated Mg-based scaffolds can offer adequate pore size to support cell ingrowth.

### 3.2. The Release of Mg^2+^ Ions

**Figure 2:**
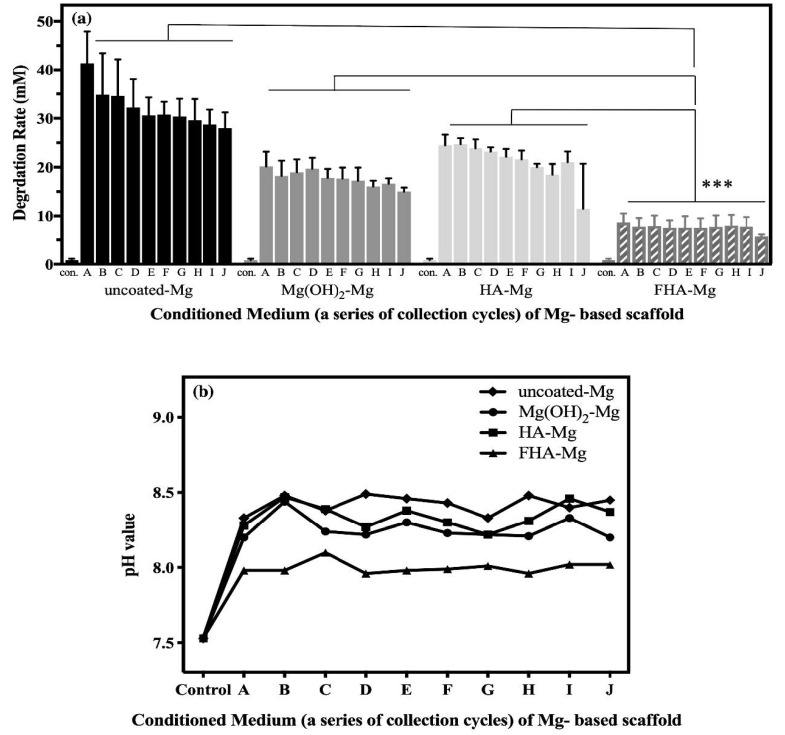
(**a**) The concentrations of released Mg^2+^ ions. Values shown are mean ± standard error (*n* = 3). *** *p* < 0.001 between FHA-Mg group and each of the other three groups. (**b**) The pH values of different Mg^2+^ conditioned media.

Figure 2 illustrates the variation of Mg^2+^ concentrations and pH values of the Mg^2+^ conditioned media A–J. The degradation rates, represented by the Mg^2+^ concentrations in conditioned media, of uncoated Mg, Mg(OH)_2_-Mg, HA-Mg, and FHA-Mg scaffolds were evaluated by collections once every 3 days for 30 days. The pH values of all the collected Mg^2+^ conditioned media are between 7.96 and 8.49, as shown in Figure 2b. Regarding the released Mg^2+^ concentration (see Figure 2a), the average Mg^2+^ concentration of uncoated Mg (AZ91) conditioned media is 32.1 mM (ranging between 28.0 and 41.4 mM). As for the hydrothermally coated Mg-based scaffolds, the average Mg^2+^ concentrations of Mg(OH)_2_-Mg, HA-Mg, and FHA-Mg conditioned media are about 17.7 mM (15.0–20.2 mM), 21.1 mM (11.5–24.7 mM), and 7.6 mM (5.7–8.7 mM), respectively. It is observed that Mg^2+^ ions leached from the FHA-Mg scaffolds are significantly reduced compared to the uncoated Mg, Mg(OH)_2_-Mg, and HA-Mg scaffolds (*p* < 0.001). Particularly, the FHA coating displays a good control of the Mg^2+^ ion release and the accumulated Mg^2+^ concentration after a three-day incubation of the sample is only 7.6 mM. Whilea high degradation rate and unstable pH value are observed in conditioned media (A–C) initially collected from uncoated and coated Mg scaffolds, both values become stable starting from the collection of conditioned medium D, which is after a 9-day sample incubation.

### 3.3. Effect of Released Mg^2+^ ions on Cell Viability

**Figure 3:**
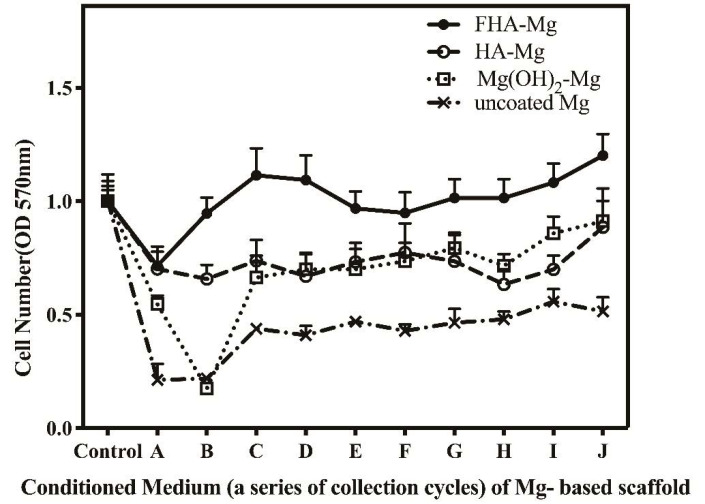
Relative cell number measured by 3-(4,5-dimethylthiazol-2-yl)-2,5-diphenyltetrazolium bromide (MTT) assay for hMSCs cultured in conditioned media collected at different time points from uncoated Mg, Mg(OH)_2_-Mg, HA-Mg, and FHA-Mg scaffolds. Values shown are mean ± standard error (*n* = 3).

**Figure 4:**
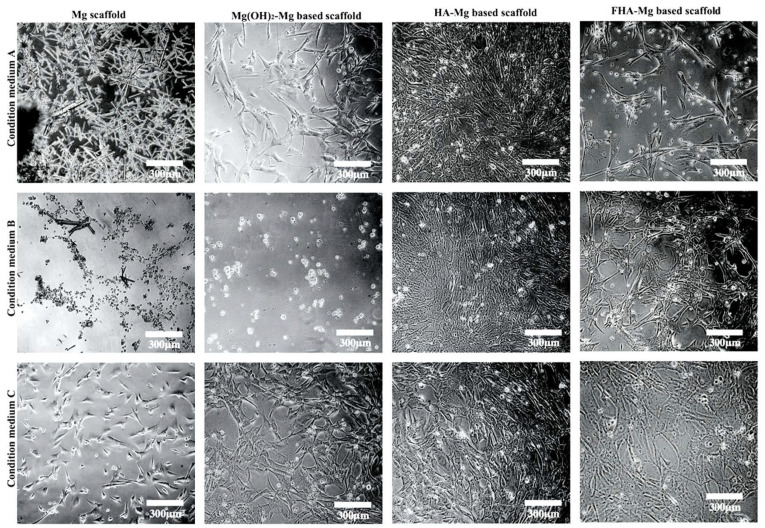
Images of hMSCs cultured for 2 days in conditioned media collected at different time points from uncoated Mg, Mg(OH)_2_-Mg, HA-Mg, and FHA-Mg scaffolds.

In order to investigate if hMSCs can survive in the chemical environment of degradation products, we examined the viability of hMSCs cultured in conditioned media A–J collected from the incubation of uncoated Mg, Mg(OH)_2_-Mg, HA-Mg, and FHA-Mg scaffolds. As determined by the MTT assay, for each alloy sample, the measured cell viability is relatively low in conditioned media A–C and becomes higher and stable after day 9 (Figure 3, D–J). As expected, hMSCs show the lowest viability when they are cultured in the conditioned media collected from uncoated Mg incubation (Figure 3). Phase contrast images of hMSCs cultured in conditioned media A–C for 2 days concur with the MTT assay results (Figure 4). In the conditioned medium A of uncoated Mg, almost no hMSCs are found, but a large number of crystalline needle-like magnesium hydroxide (Mg(OH) _2_) precipitations are generally observed. These precipitations become gradually less after a longer incubation of uncoated Mg, as shown in the conditioned media B and C (Figure 4, left panel). No apparent precipitation of magnesium hydroxide is seen in the conditioned media collected from the other three Mg-based alloys. Particularly, the cell viabilities of hMSCs cultured in conditioned media D–J collected from FHA-Mg scaffolds are significantly higher than those of cells cultured in conditioned media D–J collected from Mg(OH)_2_-Mg or HA-Mg scaffolds (*p* < 0.001). The results shown in Figure 2 and Figure 3 indicate that incubation of Mg-based scaffolds for several days (9 days in this study) can remove a large amount of initially released Mg^2+^ ions.

### 3.4. hMSC Morphology and Proliferation on FHA-Mg Scaffolds

**Figure 5:**
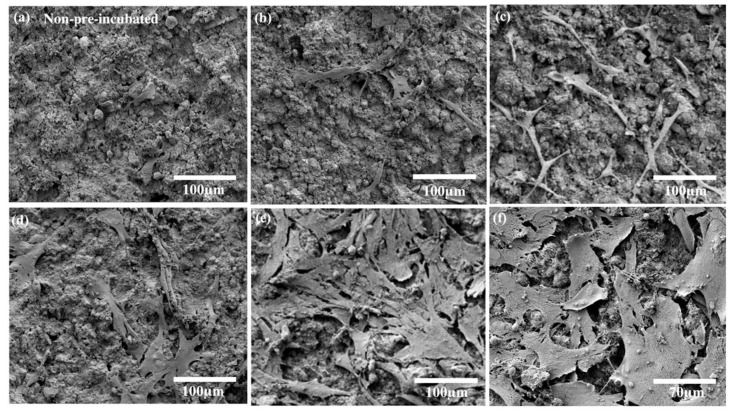
SEM micrographs of the hMSC morphology cultured on FHA-Mg-based scaffolds (**a**) without pre-incubation, and after (**b**) 3, (**c**) 5, (**d**) 7, and (**e**,**f**) 14 days of incubation. Image f is an SEM micrograph with higher magnification.

**Figure 6:**
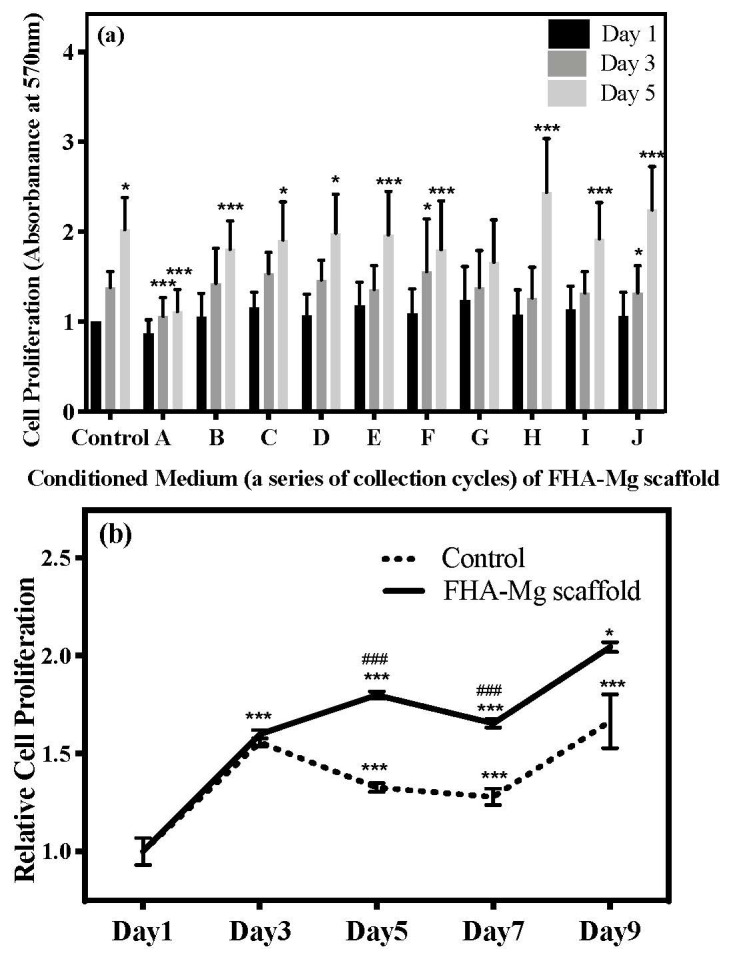
(**a**) AlamarBlue^®^ assay of hMSC proliferation on a culture dish in the conditioned media collected from FHA-Mg scaffolds at sequential time points. hMSCs cultured on a culture dish in maintenance culture medium were the control group. Values shown are mean ± standard error (*n* = 3). * *p* < 0.05, *** *p* < 0.001 as compared to day 1 of each conditioned medium. (**b**) hMSC proliferation on a culture dish (control) and on 9-day pre-incubated FHA-Mg scaffolds in maintenance culture medium. * *p* < 0.05, *** *p* < 0.001 as compared to day 1 within each group; ### *p* < 0.001 as compared at different time points between FHA-Mg and control groups.

The cell viability data of hMSCs cultured in Mg^2+^ conditioned media, as shown in Figure 3, demonstrate that the hydrothermally synthesized FHA coating effectively reduces the excessive release of Mg^2+^ ions, and subsequent experiments, therefore, focus on hMSC proliferation and osteogenic differentiation on the FHA-Mg scaffold. The typical SEM images of the hMSCs after a 3-day cultivation on FHA-Mg scaffolds with or without pre-incubation are shown in Figure 5. Compared with hMSCs cultured on FHA-Mg scaffolds without pre-incubation (Figure 5a), cells on scaffolds pre-incubated for 7 days or 14 days displayed better spreading and proliferation rate (Figure 5d–f). As seen in Figure 5f, hMSCs developed lamellipodia and filopodia which are the main protrusions formed during mesenchymal migration. The effects of FHA-Mg conditioned media on hMSC proliferation were examined by an alamarBlue^®^ assay and the results are shown in Figure 6a. Control cells, which were cultured on culture dish in complete culture medium, displayed an increase in proliferation with a significant increase at day 5 (*p* < 0.01). Compared with the control, cells cultured on a culture dish in conditioned media collected from FHA-Mg scaffolds showed a similar trend in proliferation, indicating that the extra Mg^2+^ ions in FHA-Mg conditioned media hardly inhibit hMSC proliferation. The proliferation of hMSCs on pre-incubated FHA-Mg scaffolds was further examined to clarify the surface structure dependence of hMSC proliferation. As shown in Figure 6b, compared with hMSCs cultured on a culture dish, cells cultured on the FHA-Mg scaffold displayed a higher proliferation rate. In addition, the proliferation rate of the FHA-Mg group reached 200% after 9 days (*p* < 0.01).

### 3.5. Osteogenic Differentiation of hMSCs on FHA-Mg Scaffolds

**Figure 7:**
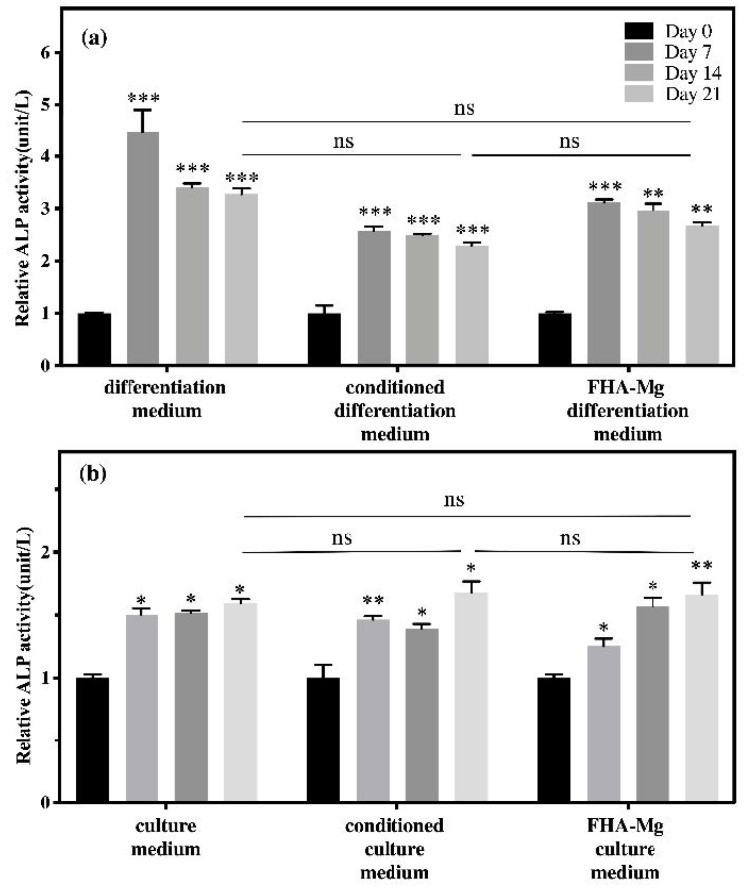
Alkaline phosphatase (ALP) activity of hMSCs cultured on culture dishes (left and middle column groups) and FHA-Mg scaffolds (right column group) in (**a**) osteogenic induction media and (**b**) culture media at 0, 7, 14, and 21 days. The relative ALP activities under various conditions were normalized to that at day 0. Conditioned differentiation or culture media used in this experiment were collected from the FHA-Mg sample incubation in constantly replenished fresh media once every 3 days over a 21-day period. Values shown are mean ± standard error (*n* = 3). * *p* < 0.05, ** *p* < 0.01, *** *p* < 0.001 as compared to day 0. N.S.: no significant difference between groups at day 21.

**Figure 8:**
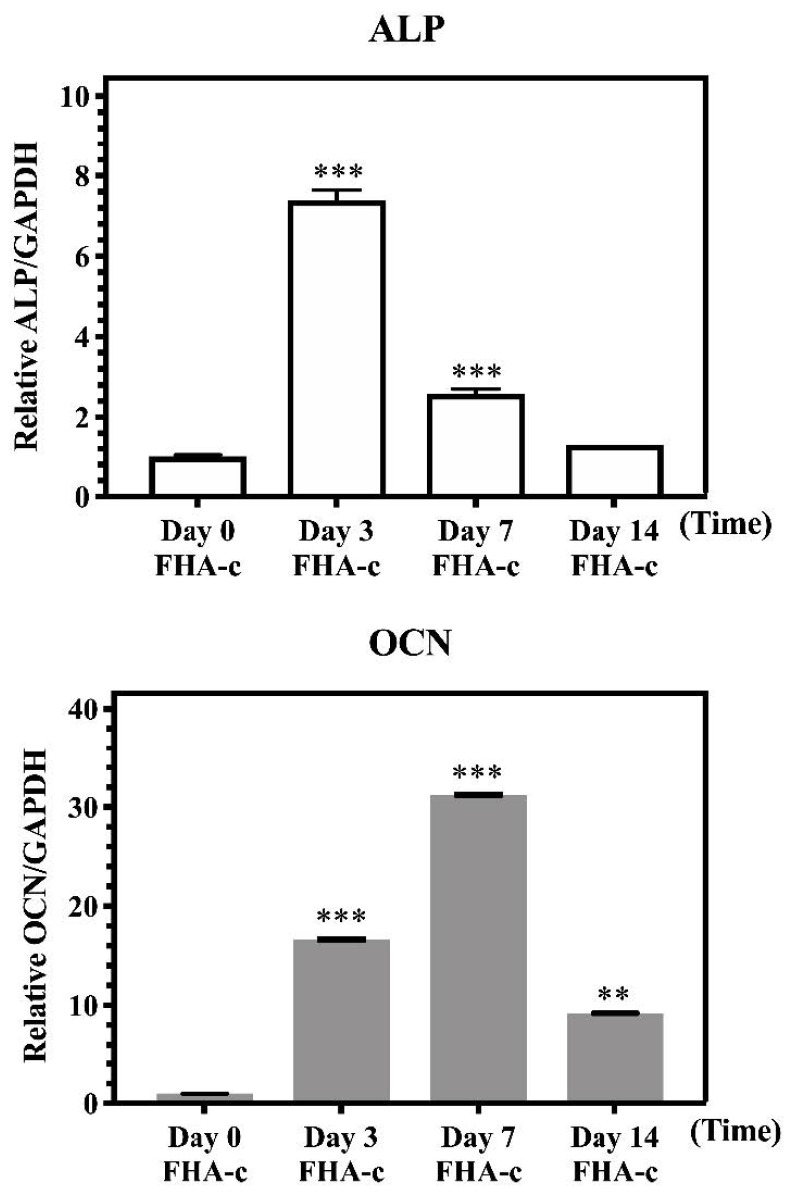
qPCR analysis of osteogenic (ALP and OCN) gene expression in hMSCs cultured on FHA-Mg scaffolds under osteogenic differentiation for 0, 3, 7, and 14 days. Relative gene expression indicates fold change of gene expression in comparison with that of undifferentiated hMSCs at day 0. All data are expressed as mean ± standard deviation (SD). Values shown are mean ± standard error (*n* = 3). ** *p* < 0.01, *** *p* < 0.001 as compared to day 0.

To identify whether FHA-Mg scaffolds affect the osteogenic differentiation of hMSCs, ALP activities of hMSCs under different culture conditions (on a culture dish or on FHA scaffolds, with or without FHA-Mg conditioned medium, and with or without osteogenic induction medium) were measured for 0, 7, 14, and 21 days (Figure 7). As shown in Figure 7a, when cultured on a culture dish (left column group) or FHA-Mg scaffolds (right column group), the ALP activity of hMSCs under osteogenic induction significantly increased at day 7 and then dropped slightly at day 14 and day 21 (*p* < 0.001). For hMSCs cultured on a culture dish with the conditioned osteogenic induction medium (middle column group), the average ALP activities at day 7 were approximately 30% and 20% lower than those of hMSCs cultured with regular osteogenic induction medium on a culture dish and FHA-Mg scaffolds, respectively. However, at day 21, there were no significant differences in ALP activity between these three groups. For hMSCs cultured on a culture dish in non-induced culture medium (left column group) or conditioned culture medium (middle column group), no significant differences were found for all the time points (Figure 7b). The results shown in Figure 7 indicate a harmless effect of magnesium-rich conditioned medium on osteogenic differentiation. Interestingly, it is noted that hMSCs cultured on FHA-Mg scaffolds (right column group) in culture medium without osteogenic induction factors demonstrated a trend of increased ALP activity with time. To further verify the positive effect of FHA-Mg scaffolds on the osteogenic differentiation of hMSCs, as shown in Figure 7, osteoblast gene expression (ALP and OCN) was also investigated by qPCR analysis 0, 3, 7, and 14 days after osteogenic induction. The analysis data are presented in Figure 8. An approximately seven-fold increase in the expression of ALP was observed at day 3. In addition, for OCN, an approximately twenty-fold and higher increase in the expression at day 3 and day 7 was observed (*p* < 0.001). Taken together, the results obtained from the ALP assay and qPCR suggest that FHA-Mg scaffolds have potential as a guided differentiation tool for directing hMSCs into osteoblast-like cells.

## 4. Discussion

In general, the surface modification of Mg-based alloys is aimed at reducing the degradation rate, maintaining mechanical strength, and providing good surface biocompatibility [14,15,29]. In this study, three biodegradable Mg-based scaffolds, Mg(OH)_2_-Mg, HA-Mg, and FHA-Mg, were fabricated and evaluated for their porosity, surface roughness, and degradation rate. Related research indicated that the Mg-based scaffold with a calcium phosphate (CaP) surface coating has low porosity [16], and its pore size is accessible for tissue engineering because the average diameter of hMSCs is about 18–30 μm. We have previously demonstrated that the FHA-Mg scaffold has a more compact structure than Mg(OH)_2_-Mg and HA-Mg scaffolds [13]. Among these samples, while the overall pore size, porosity, and surface roughness of Mg(OH)_2_-Mg and HA-Mg were not significantly different, FHA-Mg displayed the largest pore size but lowest porosity and surface roughness (Figure 1 and Table 3). Previous studies done by Witte et al. indicate that Mg-based implants can be used for the replacement of subchondral bone plates [5,51]. The advantages of Mg implants for osseous growth have been validated in various clinical indication-orientated animal models. However, unlike bioinert, non-degradable implant materials such as titanium and stainless steel, the initially high degradation rate of Mg implants imposes restrictions on osseous growth [52,53].

In the present study, the severe degradation rate of magnesium was reduced and the release of Mg^2+^ ions was effectively controlled by using an optimal protective surface layer of the hydrothermal FHA coating deposited on the Mg-based scaffold [13]. Because the degradation rate determination by weight loss may also count the weight loss of the coating, the released Mg^2+^ concentration and pH value were measured. Based on the ICP-AES analysis, the FHA-Mg sample displayed the lowest degradation rate compared to the uncoated Mg, Mg(OH)_2_-Mg, and HA-Mg samples (Figure 2). The decrease in the degradation rate by the deposition of the hydrothermal FHA coating could be related to the substitution of F^−^ ions which significantly increase coating density as an effective protective layer and lead to high crystallinity and reduction in the amount of porosity [36,39,40,54]. This result is consistent with a previous investigation of different apatite coatings on Mg-Zn alloy, which showed that these coatings decreased the corrosion rate of Mg-Zn alloy, leading to less change in the pH value and Mg^2+^ release [37,38,43].

The in vitro viability of hMSCs cultured in conditioned culture medium was determined by an MTT assay and microscope observation. As shown in Figure 3, for uncoated Mg, Mg(OH)_2_-Mg, and HA-Mg conditioned media collected from day 9 (labeled as C) to day 30 (labeled as J), the relative cell numbers were approximately 50%, 70%, and 70%, respectively, significantly lower than the FHA-Mg group (~120%) (*p* < 0.001). This cytotoxic effect is mainly due to the high Mg^2+^ concentrations and pH values created by the rapid degradation rates of Mg-based alloys, except FHA-Mg. As shown in Figure 4, apparently, hMSCs grown in Mg(OH)_2_-Mg, HA-Mg, or FHA-Mg conditioned media collected at day 9 (labeled as C) exhibited a larger spreading area and pseudopodia elongation than cells grown in those media collected at day 3 and day 6 (labeled as A and B). Despite a fairly static Mg^2+^ concentration, ~7.6 mM, in FHA-Mg conditioned medium collected at various time points (Figure 2), hMSCs maintained better viability in conditioned media collected from day 9 to day 30 than in those collected at day 3 and day 6, indicating a possible high concentration of calcium and/or phosphate ions released from the dissolved HA during the early stage of sample immersion. A recent study has shown that Ca^2+^ and PO_4_^3-^ concentrations equal to or greater than 32 mM and 16 mM, respectively, are cytotoxic for murine mesenchymal stem cells [55]. Since we focused on cell–material interactions, in this study, FHA-Mg scaffolds were pre-incubated in constantly replenished fresh culture medium for 9 days before cell seeding and subsequent in vitro tests. This pretreatment is presumed to maintain a steady corrosion rate and ion release of the FHA-Mg samples before their use in vitro or in vivo.

The in vitro biocompatibility of hMSCs in contact with the FHA-Mg scaffolds was determined by cell attachment and proliferation tests. It is noteworthy that cells attached and grew very well on the pre-incubated FHA-Mg scaffolds and no obvious toxic effects were observed (Figure 5 and Figure 6b). The presence of calcium and phosphate on HA and FHA coating surfaces has been reported to promote protein adsorption and osteoblast adhesion [12,54,56]. Cells grown on FHA-Mg scaffolds pre-incubated for 7 and 14 days (Figure 5d,e) exhibited better attachment and higher cell density than on those pre-incubated for 0, 3, and 5 days (Figure 5a–c). Cells cultured in FHA-Mg conditioned media collected from day 6 to day 30 displayed similar growth trend to the control cells which were in the culture medium (Figure 6a) (*p* < 0.01). This result corresponds with our viability data which show hMSCs are 90–120% viable in FHA-Mg conditioned media collected from day 6 to day 30 (Figure 3). Although the Mg^2+^ concentration in the FHA-Mg conditioned culture medium (~7.6 mM) is much higher than that in hMSC culture medium (~1 mM), no harmful effect on hMSC proliferation is observed, as shown in Figure 6a. Therefore, we demonstrate that pre-incubated FHA-Mg scaffolds display a suitable physiochemical environment and surface biocompatibility for hMSC attachment and proliferation, which is consistent with the lower degradation rate of FHA-Mg material and higher cell viability observed in FHA-Mg conditioned media collected at and after day 9, as shown in Figure 2 and Figure 3.

We have also demonstrated in this report that FHA-Mg scaffolds are suitable biomaterials for hMSC osteogenic differentiation (determined by ALP assay and qPCR analysis). It is important to note that, in the culture medium without osteogenic induction factors, the relative ALP activity of hMSCs cultured on FHA-Mg scaffolds increased approximately 66% over 21 days. (right column group in Figure 7b). Many studies have investigated the effects of various Mg^2+^ concentrations on the osteogenic differentiation of MSCs [57,58]. For example, a high extracellular Mg^2+^ concentration (≥1.3 mM) has been reported to significantly inhibit extracellular matrix mineralization in human bone marrow-derived mesenchymal stem cells (hBMSCs) [59,60]. On the contrary, Yoshizawa et al. reported that compared with the hBMSCs cultured in medium containing 0.8 mM MgSO_4_, a higher cell proliferation rate and extracellular mineralization were induced by 5–10 mM MgSO_4_ [25]. Different conclusions between these studies might be attributed to different sources of magnesium ions, cell types, and cell culture conditions. Future studies are needed to identify the optimal Mg^2+^ conditions for promoting osteogenic differentiation and another for the cell motility of hMSCs. As seen in Figure 7, since the existence of 5.8–7.6 mM Mg^2+^ in FHA-Mg conditioned media has no harmful effect on osteogenic differentiation, the FHA coating with nano-scaled needle-like surface features may play a functional role in osteogenic differentiation. Based on this observation, one can expect a feasible use of FHA-Mg scaffolds in vivo. In vivo experiments, such as the use of magnesium-based alloys as surgical suture anchors for bone repair, are carried out in our lab. The degradability and biocompatibility of the Mg-based scaffolds during the process of bone repair are examined. The results will enhance our understanding of the detailed nature of Mg-based scaffolds under in vivo conditions and elucidate the possible use of these materials for bone tissue engineering.

## 5. Conclusions

In this study, we demonstrated that FHA-Mg scaffolds display controlled Mg^2+^ release and good surface biocompatibility for the in vitro proliferation and osteogenic differentiation of hMSCs. These positive results are possibly attributable to the surface modification of Mg alloys. With these favorable outcomes, FHA-Mg scaffolds are promising biomaterials for orthopedic applications.

## Figures and Tables

**Table 1:** Collection time period of Mg^2+^ conditioned media from Mg-based scaffolds.

Days	0–3	4–6	7–9	10–12	13–15	16–18	19–21	22–24	25–27	28–30
Uncoated Mg conditioned medium	A	B	C	D	E	F	G	H	I	J
Mg(OH)_2_-Mg conditioned medium	A	B	C	D	E	F	G	H	I	J
HA-Mg conditioned medium	A	B	C	D	E	F	G	H	I	J
FHA-Mg conditioned medium	A	B	C	D	E	F	G	H	I	J

**Table 2:** Primers used in qPCR to evaluate the osteogenic differentiation of human mesenchymal stem cells (hMSCs).

Gene		Size (bp)	Sequences (5′ to 3′)
Glyceraldehyde 3-phosphate dehydrogenase (GAPDH)	forward	71	GAA GGT GAA GGT CGG AGT CAA C
	reverse		CAG AGT TAA AAG CAG CCC TGG T
Alkaline phosphatase (ALP)	forward	476	ACG TGG CTA AGA ATG TCA TC
	reverse		CTG GTA GGC GAT GTC CTT A
Osteocalcin (OCN)	forward	315	CAT GAG AGC CCT CAC A
	reverse		AGA GCG ACA CCC TAG AC

**Table 3:** Porosity and surface roughness of the uncoated and hydrothermally coated Mg-based scaffolds.

	Uncoated Mg Scaffold	Mg(OH)_2_-Mg Scaffold	HA-Mg Scaffold	FHA-Mg Scaffold
**Pore size (μm)**	N/A	48.5 ± 1.6	45.6 ± 1.0	61.3 ± 0.6
**Porosity (vol.%)**	N/A	18.4 ± 1.6	13.1 ± 0.9	8.5 ± 0.5
**Roughness (μm)**	0.09 ± 0.03	9.46 ± 0.92	8.30 ± 1.74	4.12 ± 0.68

## Data Availability

The data presented in this study are available upon reasonable request from the corresponding author.

## References

[1] Amini A.R., Laurencin C.T., Nukavarapu S.P. (2012). Bone tissue engineering: recent advances and challenges. Crit. Rev. Biomed. Eng..

[2] Bose S., Roy M., Bandyopadhyay A. (2012). Recent advances in bone tissue engineering scaffolds. Trends Biotechnol..

[3] Jiang Y., Wang D., Blocki A., Tuan R.S., Lanza R., Langer R., Vacanti J.P., Atala A. (2020). Chapter 49—Mesenchymal stem cells in musculoskeletal tissue engineering. Principles of Tissue Engineering.

[4] Zhao D., Witte F., Lu F., Wang J., Li J., Qin L. (2017). Current status on clinical applications of magnesium-based orthopaedic implants: A review from clinical translational perspective. Biomaterials.

[5] Witte F., Kaese V., Haferkamp H., Switzer E., Meyer-Lindenberg A., Wirth C.J., Windhagen H. (2005). In vivo corrosion of four magnesium alloys and the associated bone response. Biomaterials.

[6] Walker J., Shadanbaz S., Woodfield T.B., Staiger M.P., Dias G.J. (2014). Magnesium biomaterials for orthopedic application: A review from a biological perspective. J. Biomed. Mater. Res. B Appl. Biomater..

[7] Han H.-S., Loffredo S., Jun I., Edwards J., Kim Y.-C., Seok H.-K., Witte F., Mantovani D., Glyn-Jones S. (2019). Current status and outlook on the clinical translation of biodegradable metals. Mater. Today.

[8] Angrisani N., Reifenrath J., Zimmermann F., Eifler R., Meyer-Lindenberg A., Vano-Herrera K., Vogt C. (2016). Biocompatibility and degradation of LAE442-based magnesium alloys after implantation of up to 3.5 years in a rabbit model. Acta Biomater..

[9] Waizy H., Diekmann J., Weizbauer A., Reifenrath J., Bartsch I., Neubert V., Schavan R., Windhagen H. (2014). In vivo study of a biodegradable orthopedic screw (MgYREZr-alloy) in a rabbit model for up to 12 months. J. Biomater. Appl..

[10] Yang H., Jia B., Zhang Z., Qu X., Li G., Lin W., Zhu D., Dai K., Zheng Y. (2020). Alloying design of biodegradable zinc as promising bone implants for load-bearing applications. Nat. Commun..

[11] Goodman S.B., Yao Z., Keeney M., Yang F. (2013). The future of biologic coatings for orthopaedic implants. Biomaterials.

[12] Su Y., Cockerill I., Zheng Y., Tang L., Qin Y.-X., Zhu D. (2019). Biofunctionalization of metallic implants by calcium phosphate coatings. Bioact. Mater..

[13] Wang S.-H., Yang C.-W., Lee T.-M. (2016). Evaluation of microstructural features and in vitro biocompatibility of hydrothermally coated fluorohydroxyapatite on AZ80 Mg alloy. Ind. Eng. Chem. Res..

[14] Wang Z., Wang X., Pei J., Tian Y., Zhang J., Jiang C., Huang J., Pang Z., Cao Y., Wang X. (2020). Degradation and osteogenic induction of a SrHPO4-coated Mg–Nd–Zn–Zr alloy intramedullary nail in a rat femoral shaft fracture model. Biomaterials.

[15] Razavi M., Fathi M., Savabi O., Tayebi L., Vashaee D. (2020). Biodegradable Magnesium Bone Implants Coated with a Novel Bioceramic Nanocomposite. Materials.

[16] Liu C., Wang J., Gao C., Wang Z., Zhou X., Tang M., Yu K., Deng Y. (2020). Enhanced osteoinductivity and corrosion resistance of dopamine/gelatin/rhBMP-2–coated β-TCP/Mg-Zn orthopedic implants: An in vitro and in vivo study. PLoS ONE.

[17] Xu T., He X., Chen Z., He L., Lu M., Ge J., Weng J., Mu Y., Duan K. (2019). Effect of magnesium particle fraction on osteoinduction of hydroxyapatite sphere-based scaffolds. J. Mater. Chem. B.

[18] Deligianni D.D., Katsala N.D., Koutsoukos P.G., Missirlis Y.F. (2001). Effect of surface roughness of hydroxyapatite on human bone marrow cell adhesion, proliferation, differentiation and detachment strength. Biomaterials.

[19] Mohd Daud N., Sing N.B., Yusop A.H., Abdul Majid F.A., Hermawan H. (2014). Degradation and in vitro cell–material interaction studies on hydroxyapatite-coated biodegradable porous iron for hard tissue scaffolds. J. Orthop. Transl..

[20] Willumeit-Römer R. (2019). The Interface Between Degradable Mg and Tissue. JOM.

[21] Li X., Liu X., Wu S., Yeung K.W.K., Zheng Y., Chu P.K. (2016). Design of magnesium alloys with controllable degradation for biomedical implants: From bulk to surface. Acta Biomater..

[22] Sojka J.E., Weaver C.M. (1995). Magnesium supplementation and osteoporosis. Nutr. Rev..

[23] DiNicolantonio J.J., O’Keefe J.H., Wilson W. (2018). Subclinical magnesium deficiency: A principal driver of cardiovascular disease and a public health crisis. Open Heart.

[24] Grigolato R., Pizzi N., Brotto M.C., Corrocher G., Desando G., Grigolo B. (2015). Magnesium-enriched hydroxyapatite as bone filler in an ameloblastoma mandibular defect. Int. J. Clin. Exp. Med..

[25] Yoshizawa S., Brown A., Barchowsky A., Sfeir C. (2014). Magnesium ion stimulation of bone marrow stromal cells enhances osteogenic activity, simulating the effect of magnesium alloy degradation. Acta Biomater..

[26] Wu Y.F., Wang Y.M., Jing Y.B., Zhuang J.P., Yan J.L., Shao Z.K., Jin M.S., Wu C.J., Zhou Y. (2017). In vivo study of microarc oxidation coated biodegradable magnesium plate to heal bone fracture defect of 3mm width. Colloids Surf. B Biointerfaces.

[27] Yun Y., Dong Z., Yang D., Schulz M.J., Shanov V.N., Yarmolenko S., Xu Z., Kumta P., Sfeir C. (2009). Biodegradable Mg corrosion and osteoblast cell culture studies. Mater. Sci. Eng. C.

[28] Roy M.E., Nishimoto S.K. (2002). Matrix Gla protein binding to hydroxyapatite is dependent on the ionic environment: Calcium enhances binding affinity but phosphate and magnesium decrease affinity. Bone.

[29] Tang J., Wang J., Xie X., Zhang P., Lai Y., Li Y., Qin L. (2013). Surface coating reduces degradation rate of magnesium alloy developed for orthopaedic applications. J. Orthop. Transl..

[30] Tsao Y.-T., Shih Y.-Y., Liu Y.-A., Liu Y.-S., Lee O.K. (2017). Knockdown of SLC41A1 magnesium transporter promotes mineralization and attenuates magnesium inhibition during osteogenesis of mesenchymal stromal cells. Stem Cell Res. Ther..

[31] Yue J., Jin S., Gu S., Sun R., Liang Q. (2019). High concentration magnesium inhibits extracellular matrix calcification and protects articular cartilage via Erk/autophagy pathway. J. Cell Physiol..

[32] Huang B., Yuan Y., Li T., Ding S., Zhang W., Gu Y., Liu C. (2016). Facilitated receptor-recognition and enhanced bioactivity of bone morphogenetic protein-2 on magnesium-substituted hydroxyapatite surface. Sci. Rep..

[33] Omidi M., Agha N.A., Müller A., Feyerabend F., Helmholz H., Willumeit-Römer R., Schlüter H., Luthringer-Feyerabend B. (2020). Investigation of the impact of magnesium versus titanium implants on protein composition in osteoblast by label free quantification. Metallomics.

[34] Tian P., Liu X. (2015). Surface modification of biodegradable magnesium and its alloys for biomedical applications. Regen Biomater..

[35] Dorozhkin S.V. (2014). Calcium orthophosphate coatings on magnesium and its biodegradable alloys. Acta Biomater..

[36] Chen Y., Miao X. (2004). Effect of fluorine addition on the corrosion resistance of hydroxyapatite ceramics. Ceram. Int..

[37] Bakhsheshi-Rad H., Hamzah E., Daroonparvar M., Ebrahimi-Kahrizsangi R., Medraj M. (2014). In-vitro corrosion inhibition mechanism of fluorine-doped hydroxyapatite and brushite coated Mg–Ca alloys for biomedical applications. Ceram. Int..

[38] Zhao C., Hou P., Ni J., Han P., Chai Y., Zhang X. (2016). Ag-incorporated FHA coating on pure Mg: degradation and in vitro antibacterial properties. Acs Appl. Mater. Interfaces.

[39] Ebrahimi-Kahrizsangi R., Nasiri-Tabrizi B., Chami A. (2011). Characterization of single-crystal fluorapatite nanoparticles synthesized via mechanochemical method. Particuology.

[40] Bakhsheshi-Rad H., Hamzah E., Daroonparvar M., Yajid M., Kasiri-Asgarani M., Abdul-Kadir M., Medraj M. (2014). In-vitro degradation behavior of Mg alloy coated by fluorine doped hydroxyapatite and calcium deficient hydroxyapatite. Trans. Nonferrous Met. Soc. China.

[41] Ansari Z., Kalantar M., Kharaziha M., Ambrosio L., Raucci M.G. (2020). Polycaprolactone/fluoride substituted-hydroxyapatite (PCL/FHA) nanocomposite coatings prepared by in-situ sol-gel process for dental implant applications. Prog. Org. Coat..

[42] Zhang X., Wang B., Ma L., Xie L., Yang H., Li Y., Wang S., Qiao H., Lin H., Lan J. (2020). Chemical stability, antibacterial and osteogenic activities study of strontium-silver co-substituted fluorohydroxyapatite nanopillars: A potential multifunctional biological coating. Ceram. Int..

[43] Shen S., Cai S., Bao X., Xu P., Li Y., Jiang S., Xu G. (2018). Biomimetic fluoridated hydroxyapatite coating with micron/nano-topography on magnesium alloy for orthopaedic application. Chem. Eng. J..

[44] Liu S., Zhou H., Liu H., Ji H., Fei W., Luo E. (2019). Fluorine-contained hydroxyapatite suppresses bone resorption through inhibiting osteoclasts differentiation and function in vitro and in vivo. Cell Prolif..

[45] Razavi M., Fathi M., Savabi O., Boroni M. (2012). A review of degradation properties of Mg based biodegradable implants. Res. Rev. Mater. Sci. Chem.

[46] Wang Y., Li X., Chen M., Zhao Y., You C., Li Y., Chen G. (2019). In Vitro and in Vivo Degradation Behavior and Biocompatibility Evaluation of Microarc Oxidation-Fluoridated Hydroxyapatite-Coated Mg–Zn–Zr–Sr Alloy for Bone Application. ACS Biomater. Sci. Eng..

[47] Ratnayake J.T., Mucalo M., Dias G.J. (2017). Substituted hydroxyapatites for bone regeneration: A review of current trends. J. Biomed. Mater. Res. Part B Appl. Biomater..

[48] Wallin R.F., Arscott E. (1998). A practical guide to ISO 10993-5: Cytotoxicity. Med. Device Diagn. Ind..

[49] (2012). Biological Evaluation of Medical Devices—Part 12: Sample Preparation and Reference Materials. Association for the Advancement of Medical Instrumentation.

[50] Ge J., Guo L., Wang S., Zhang Y., Cai T., Zhao R.C.H., Wu Y. (2014). The Size of Mesenchymal Stem Cells is a Significant Cause of Vascular Obstructions and Stroke. Stem Cell Rev. Rep..

[51] Witte F., Fischer J., Nellesen J., Crostack H.A., Kaese V., Pisch A., Beckmann F., Windhagen H. (2006). In vitro and in vivo corrosion measurements of magnesium alloys. Biomaterials.

[52] Kawamura N., Nakao Y., Ishikawa R., Tsuchida D., Iijima M. (2020). Degradation and Biocompatibility of AZ31 Magnesium Alloy Implants in vitro and in vivo: A Micro-Computed Tomography Study in Rats. Materials.

[53] Yu W., Chen D., Ding Z., Qiu M., Zhang Z., Shen J., Zhang X., Zhang S., He Y., Shi Z. (2016). Synergistic effect of a biodegradable Mg–Zn alloy on osteogenic activity and anti-biofilm ability: an in vitro and in vivo study. RSC Adv..

[54] Razavi M., Fathi M., Savabi O., Vashaee D., Tayebi L. (2015). In vivo assessments of bioabsorbable AZ91 magnesium implants coated with nanostructured fluoridated hydroxyapatite by MAO/EPD technique for biomedical applications. Mater. Sci. Eng. C Mater. Biol. Appl..

[55] Ali Akbari Ghavimi S., Allen B.N., Stromsdorfer J.L., Kramer J.S., Li X., Ulery B.D. (2018). Calcium and phosphate ions as simple signaling molecules with versatile osteoinductivity. Biomed. Mater..

[56] Feng B., Weng J., Yang B.C., Qu S.X., Zhang X.D. (2004). Characterization of titanium surfaces with calcium and phosphate and osteoblast adhesion. Biomaterials.

[57] Qi T., Weng J., Yu F., Zhang W., Li G., Qin H., Tan Z., Zeng H. (2020). Insights into the Role of Magnesium Ions in Affecting Osteogenic Differentiation of Mesenchymal Stem Cells. Biol. Trace Elem. Res..

[58] Luthringer B.J., Willumeit-Romer R. (2016). Effects of magnesium degradation products on mesenchymal stem cell fate and osteoblastogenesis. Gene.

[59] Zhang L., Yang C., Li J., Zhu Y., Zhang X. (2014). High extracellular magnesium inhibits mineralized matrix deposition and modulates intracellular calcium signaling in human bone marrow-derived mesenchymal stem cells. Biochem. Biophys. Res. Commun..

[60] Zhang J., Tang L., Qi H., Zhao Q., Liu Y., Zhang Y. (2019). Dual Function of Magnesium in Bone Biomineralization. Adv. Healthc. Mater..

